# Basis for Cumulative Cultural Evolution in Chimpanzees: Social Learning of a More Efficient Tool-Use Technique

**DOI:** 10.1371/journal.pone.0055768

**Published:** 2013-01-30

**Authors:** Shinya Yamamoto, Tatyana Humle, Masayuki Tanaka

**Affiliations:** 1 Primate Research Institute, Kyoto University, Inuyama, Aichi, Japan; 2 School of Anthropology and Conservation, University of Kent, Canterbury, Kent, United Kingdom; 3 Wildlife Research Center, Kyoto University, Sakyo-ku, Kyoto, Japan; Illinois State University, United States of America

## Abstract

**Background:**

The evidence for culture in non-human animals has been growing incrementally over the past two decades. However, the ability for cumulative cultural evolution, with successive generations building on earlier achievements, in non-human animals remains debated. Faithful social learning of incremental improvements in technique is considered to be a defining feature of human culture, differentiating human from non-human cultures. This study presents the first experimental evidence for chimpanzees' social transmission of a more efficient tool-use technique invented by a conspecific group member.

**Methodology/Principal Findings:**

The chimpanzees were provided with a straw-tube, and spontaneously demonstrated two different techniques in obtaining juice through a small hole: “dipping” and “straw-sucking”. Both the “dipping” and “straw-sucking” techniques depended on the use of the same tool (straw-tube) for the same target (juice) accessible from exactly the same location (small hole 1 cm in diameter). Therefore the difference between “dipping” and “straw-sucking” was only in “technique”. Although the two techniques differed significantly in their efficiency, their cognitive and perceptuo-motor complexity were comparable. All five chimpanzees who initially performed the “dipping” technique switched to using the more efficient “straw-sucking” technique upon observing a conspecific or human demonstrate the more proficient alternate “straw-sucking” technique.

**Conclusions/Significance:**

The social learning mechanism involved here was clearly not local or stimulus enhancement, but imitation or emulation of a tool-use technique. When there is no biologically relevant difference in cognitive or perceptuo-motor complexity between two techniques, and when chimpanzees are dissatisfied with their own technique, chimpanzees may socially learn an improved technique upon close observation of a proficient demonstrator. This study provides important insights into the cognitive basis for cumulative culture in chimpanzees, and also suggests possible conditions in which cumulative cultural evolution could arise even in non-human animals.

## Introduction

Culture in non-human animals is one of the hottest and most debated questions within the social and biological sciences. Putative cultural variants are by definition independent of environmental or genetic differences and are maintained via social learning mechanisms [1]–[3]. Candidate examples of culture across the animal kingdom have been accumulating incrementally over the course of the past two decades [1]–[3]. However, many argue that humans are still unique in their capacity for cumulative cultural evolution, with successive generations building on earlier achievements [4]–[7]. This process depends upon faithful, high fidelity social transmission of improved, more efficient techniques. In humans, imitation and teaching are viewed as the key processes underlying cumulative cultural evolution and some researchers argue that these social learning mechanisms are absent or rare in non-human cultures [8]–[9].

Chimpanzees, one of our closest living relatives, display in the wild not only an array of different tool-use types but also tool-use techniques that vary among communities [10]–[12]. For example, when ant-dipping and gathering army ants (*Dorylus* sp.) off a tool, chimpanzees may exhibit one of two techniques: “direct mouthing” which involves the chimpanzee passing the tool through its lips, and “pull-through” which requires the chimpanzee to swipe the length of or a portion of the wand with its free hand. Chimpanzees in Taï, Côte d'Ivoire, rely predominantly on the “direct mouthing” technique [13], while in Gombe, Tanzania, the majority of chimpanzees demonstrate the “pull-through” technique [14], although Bossou chimpanzees in Guinea exhibit both [11], [15]. Although tool length does to some extent explain these differences, variations in technique cannot solely be accounted for by tool length attributes alone [16]–[17]. In addition, Goualougo chimpanzees in the Congo employ a tool set when targeting army ant nests; they use a woody tool to perforate the nest and then a more slender probing tool or wand to dip for the ants [18]. The use of a tool set in this context is thought to improve harvesting efficiency and prey exploitation over longer periods of time. Cumulative innovation in techniques is also suggested from observational and archaeological studies on chimpanzee nut-cracking [10], [19]–[20]. Complex techniques in nut-cracking involving the use of a wedge stone [21] are also plausible examples of cumulative culture. However, the mechanisms of acquisition and diffusion of these differing techniques are still not fully understood.

It remains to be examined how this variation in tool-use techniques emerges and how it is maintained within communities [22]. Although chimpanzees can learn socially a variety of behaviors including tool-use [23]–[25], the social learning mechanisms involved can sometimes be described parsimoniously as simple local or stimulus enhancement. Social learning of improved tool-use techniques requires more sophisticated mechanisms, since individuals have to differentiate two techniques which target the same goal at the same location and also involve reliance upon the same tool. Previous experimental studies have revealed that chimpanzees can socially learn different techniques [26]–[29]. However, while most of these studies have focused on the social transmission of behavioral techniques not involving tools [27]–[28], others reported social learning of two optional tool-uses whose performance differed in the target location of the tool-use action [29]. Based upon the strict criteria of same tool, same target, and same location, there is to date little experimental evidence for social transmission of tool-use techniques in non-human animals, even in chimpanzees.

It is also unclear whether or not chimpanzees are able to switch their technique to a more efficient one via social learning. Chimpanzees appear to be conservative when it comes to incorporating novel and more efficient techniques into their behavioral repertoire. Captive studies suggest that when chimpanzees become proficient at employing a particular technique, they stick to this technique even if given the opportunity to observe others demonstrating an alternate more efficient technique [30]–[31]. Many researchers therefore consider that only humans are cognitively capable of cumulative cultural evolution. However, experimental conditions or motivational factors may undermine the chimpanzees' abilities and performance. Here we present the first experimental evidence, to our knowledge, for chimpanzees' social learning of a more efficient tool-use technique in an intuitive tool-use situation, suggesting that the limitation for chimpanzees' cumulative cultural evolution might be due to ecological, social, and motivational factors rather than cognitive inabilities per se.

## Results and Discussion

**Figure 1:**
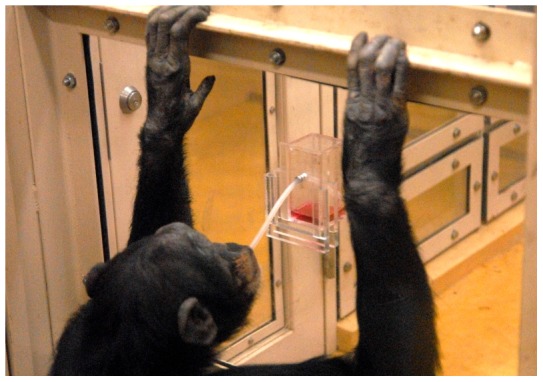
Both the “dipping” and “straw-sucking” techniques entailed the same tool (straw-tube), the same target (juice), and exactly the same location (small hole). Actually in the scene depicted in this photo, the chimpanzee thereafter retrieved the tube and licked its tip (“dipping”).

We tested nine captive chimpanzees at the Primate Research Institute, Kyoto University. Each participant was provided with an 18 cm-long silicon straw-tube. This tube could be used as a tool to obtain juice contained in a bottle externally fixed to the panel wall of the experimental booth, and accessible via a small hole (1 cm in diameter). In a pre-test examination, four of the nine chimpanzees performed the “straw-sucking” technique, while the other five adopted the “dipping” technique (Figure 1; Movie S1). The “dipping” technique was much less efficient than the “straw-sucking” technique. A chimpanzee participant could normally drink up to 50 ml of juice contained in the bottle within 30 sec (>100 ml/min) when employing the “straw-sucking” technique, while at most 20 ml during a 10 min trial (<2 ml/min) when employing the “dipping” technique. “Dipping” participants failed to innovate the “straw-sucking” technique by themselves when tested for 5 days individually, i.e., in the individual condition (10 min a day).

When we paired each of the five “dipping” participants with a “straw-sucking” conspecific non-kin demonstrator in the same booth, four of the five participants subsequently adopted the “straw-sucking” technique (Table 1). Those chimpanzees who most closely and attentively observed the demonstrator perform the alternate ‘straw-sucking’ technique switched more rapidly to using the novel technique. In the paired condition, three of the participants (Pal, Ayumu and Puchi) paid close attention to the demonstrator. They observed intently the “straw-sucking’ technique within a distance of 50 cm, and subsequently switched their technique (Movie S2). One participant, Pan, failed to observe closely the demonstrator and to show such a rapid switch in technique. Her subordinate status to the demonstrator (Pendesa) might have limited her access to the juice devices as Pendesa often occupied both juice sites available in the experimental booth. However, we never observed any displacement or agonistic interactions between the two throughout the experiment. When we placed Pan and the demonstrator separately into two adjacent booths divided by a transparent wall and each was equipped with a juice bottle device (paired 2 condition), Pan then finally switched to using the “straw-sucking” technique. Mari, who never closely attended conspecific demonstrations of the “straw-sucking” technique and consequently never learned this alternate technique, only finally switched her technique after watching consecutive demonstrations performed by a familiar human. Once the chimpanzees switched to using the “straw-sucking” technique, they never again reverted to using the less efficient “dipping” technique.

**Table 1:** Switch in tube-use techniques from “dipping” to “straw-sucking”.

	Individual					Paired 1					Individual					Paired 2				
Pal	D	D	D	D	D	DS	S	S	S	S	S	S	S	S	S					
Ayumu	D	D	D	D	D	D	DS	S	S	S	S	S	S	S	S					
Puchi	D	-	-	-	-	D	-	-	S	S	S	S	S	S	S					
Pan	D	D	-	-	-	D	-	-	-	-	D	-	-	-	-	-	S	S	S	S
Mari	-	-	-	-	-	-	-	D	-	D	-	-	D	-	-	-	D	D	D	D

Note: Individual: condition where each participant was tested individually; Paired: condition where each participant was tested with the conspecific “straw-sucking” demonstrator (1: together in a booth with two juice bottles; 2: separated in two adjacent booths each equipped with a juice bottle); D: “dipping” technique; S: “straw-sucking” technique; “DS”: firstly “dipping” technique, and then “straw-sucking” technique after observing the demonstrator's straw-sucking; -: no try; trials highlighted in grey indicate that a participant observed the demonstrator's “straw-sucking” within a distance of 50 cm.

The chimpanzees appeared to socially learn the tool-use “technique” they observed their partner perform. Both the “dipping” and “straw-sucking” techniques involved the same tool (straw-tube), the same target (juice), and exactly the same location (a hole of 1 cm in diameter drilled into the panel wall). When dipping, the chimpanzees sometimes used their mouth to manipulate and insert the tube into the bottle to dip for juice (Figure 1; Movie S1). In these cases, the form was identical to that seen in the “straw-sucking” technique. This suggests that the chimpanzees actually learned the “straw-sucking” technique by imitation or emulation rather than via simple local or stimulus enhancement. Since the straw-tube was not opaque, we could not distinguish true imitation (copying the form of the action) and emulation (replicating the visible environmental result) [32]. Even without true imitation, however, cultural differences can be maintained and transmitted as long as social learning reliably leads to the same result that was demonstrated [33].

The chimpanzee participants of this study switched their technique to a more efficient one through social learning, although previous studies [30]–[31] failed to uncover such improvements in technique. We propose two explanations for this difference in results. First, in the present study, there was no pertinent difference in perceptuo-motor and cognitive complexity between the two techniques, while there was in a previous study [30]. Both the “dipping” and “straw-sucking” techniques emerged spontaneously during the pre-test examination phase. In addition, the behavioral act of inserting the tube into the bottle was identical for the two techniques and both “dipping” and “sucking” were components of the chimpanzees' customary behavioral repertoire. Second, the chimpanzees in previous studies [30]–[31] appeared satisfied with their own existing technique, while the participants in the present study may not have been. This latter hypothesis may best explain the observed switch in technique reported here, suggesting therefore that chimpanzees do not “copy-if-better” but rather “copy-if-dissatisfied” which does not necessarily imply any sophisticated cognitive judgment of efficiency [34]. Puchi, Pan, and Mari stopped dipping during the first individual condition, which suggests that they were not satisfied with their own dipping technique. Pal and Ayumu, who continued dipping throughout the first individual condition, observed the demonstrator's sucking before she even finished drinking up the juice in the first trial of the paired condition. Therefore, Ayumu and Pal could not have readily noticed or evaluated the difference in efficiency between the two techniques. These results support the “copy-if-dissatisfied” hypothesis. The present study also demonstrates that even older chimpanzees (e.g. 41-year-old Puchi in this study) can socially learn a novel technique, if they are not satisfied with their own and are motivated enough to explore alternatives.

Chimpanzees can therefore rely on simpler cognitive mechanisms for cumulative culture than previously assumed. A study of wild chimpanzees indicated that a young chimpanzee invented and modified a novel tool-use behavior based on the existing behavioral repertoire customary of his community [35]. Although evidence for cumulative culture, i.e. the “ratchet effect”, in chimpanzees in the wild remains circumstantial and speculative [3]–[4], [7]–[9], the present study suggests that individuals can acquire improved tool-use techniques through social learning. However, who invents this novel technique may dictate its future spread and who learns thereafter from whom [23], [25], [36]. The present study also reveals that the latency to adopt the more efficient method was related to how attentive the observers were to the demonstrations. Even if a subordinate individual innovated a new technique, this novel behavior might not spread, as he or she would likely fail to act as a salient demonstrator and catalyst for the diffusion of this new behavior, albeit more efficient [29], [37]. Meanwhile, if a high-status individual or a mother acquired the new behavior, the improved behavior and technique could spread to other group members either due to the prestige of the demonstrator [37] or the inter-generational transmission from mother to offspring [38]. However, our study failed to reveal any clear effect of the non-kin demonstrator's middle-ranked status on social transmission. As previously argued [39], strong mutual tolerance to close observation, added to individual motivation, may therefore also act to promote diffusion of novel behavioral variants.

Our results also indicate that clear evidence of cumulative cultural evolution among our closest evolutionary neighbors may be constrained by other factors than their cognitive capacity. It is possible that we are currently unable to appreciate the extent of cumulative cultural evolution in chimpanzees because of the relatively short timescale of studies conducted in the wild. In addition, chimpanzees may infrequently experience ecological and/or social selective factors that would give rise to innovations reflecting improved increments in technology in combination with conditions favorable to the social transmission of the novel behavior [10]. The present study, combined with previous studies [30]–[31], suggests that chimpanzees switch technique when not satisfied with their own. Hence, necessity and opportunity appear to act as key prerequisites for cumulative cultural evolution.

## Materials and Methods

### Ethics Statement

Participants were socially housed chimpanzees at the Primate Research Institute, Kyoto University (KUPRI). The participants spend their daily life with other group members in enriched facilities [40], and had ad libitum access to water and were not food deprived. Participation in our experiment was dependant on the participant's motivation: the experimenter called the name of a participant (who was in the outdoor enclosure with other group members), and he/she could decide whether or not to take part in the study. The present study was approved by the Animal Care Committee of the Primate Research Institute of Kyoto University (approval ID; 07-1544), and the chimpanzees were tested and cared for according to “the Guide for the Care and Use of Laboratory Primates, 2^nd^ edition” produced by the ethics committee of the Primate Research Institute of Kyoto University (2002). Our procedure also followed the recommendations of the Weatherall report, “The use of non-human primates in research”.

All participants, nine chimpanzees in total, had previously taken part in a variety of perceptual and cognitive studies, including experiments which examined their honey-dipping behavior and social learning of tool-use [24], [38]. The participants (Pal: 7-year-old female, Ayumu: 7 y male, Pan: 23 y female, Mari: 31 y female, and Puchi: 41 y female) who initially demonstrated the “dipping” technique were the focal subjects of the present study. The “straw-sucking” demonstrator was a middle-ranked 30-year-old female, Pendesa, who had no kin-relationship with any of the “dipping” participants, “Dipping” was defined as inserting a flexible tube into a hole providing access to a juice reward contained in a small bottle externally affixed to the booth's panel wall, retrieving the tool, and licking the tip. “Straw-sucking” was defined as inserting the tube into the same hole and drinking the juice reward using the tube as a straw.

The chimpanzee participants were tested in an experimental booth (291 cm×192 cm, 200 cm high). In the “individual” and “paired 1” conditions, two juice bottle containers (2 m apart) and two portable translucent silicon tubes (18 cm long, 8 mm in external diameter and 3 mm in internal diameter) were available to the subjects. In the “paired 2” condition, when the subjects were tested separately in two adjacent experimental booths divided by a transparent wall (136 cm×142 cm and 155 cm×142 cm, 200 cm high), each booth was equipped with a tube and a juice container affixed to the panel wall. We first examined individually each participants' spontaneous tube-use behavior. We then selected all five “dipping” participants and one “straw-sucking” demonstrator. The participants were thereafter tested in at most 4 blocks of 5 trials in the individual and the paired conditions alternately (Table 1). We conducted one 10 min trial per day for each focal participant. We recorded the participants' behavior and interaction with three video cameras (Panasonic NV-GS150).

### Supplementary data

Supplementary data, with video clips of the two tool-use techniques and observational learning, are available as supporting materials.

## Supporting Information

Movie S1 The “dipping” technique performed by a chimpanzee Ayumu. Note that he uses his mouth to insert the tube into the bottle. In form, his technique is identical to the “straw-sucking” technique. However, instead of leaving the tube in and retrieving the juice via sucking, he removes the tube and licks the tip.
(MPG)

Movie S2 Close observation and subsequent switch in technique used. Pal (out of sight in the first view) closely observes the demonstrator, then fetches a tube from the floor (out of sight), and then proceeds to suck the remainder of the juice in the bottle container. Pal had just performed the “dipping” technique prior to observing the alternate technique being demonstrated during the same trial.
(MPG)
